# Extracranial neurogenic tumors of the head and neck^☆^^☆☆^

**DOI:** 10.1016/j.bjorl.2015.08.012

**Published:** 2015-09-08

**Authors:** Otávio Alberto Curioni, Ricardo Pires de Souza, Ana Maria da Cunha Mercante, Ana Carolina de Jesus, Alysson Pavelegeni, Rogério Aparecido Dedivitis, Abrão Rapoport

**Affiliations:** a Faculdade de Ciências Médicas de Santos, Centro Universitário Lusíada (UNILUS), São Paulo, SP, Brazil; b Department of Head and Neck Surgery and Otorhinolaryngology, Hospital Heliópolis, São Paulo, SP, Brazil; c Department of Radiology, Faculdade de Medicina, Universidade de São Paulo (USP), São Paulo, SP, Brazil; d Department of Imaging Diagnosis, Universidade Federal de São Paulo (UNIFESP), São Paulo, SP, Brazil; e Service of Pathological Anatomy, Hospital Heliópolis, São Paulo, SP, Brazil; f Larynx Group, Department of Head and Neck Surgery, Hospital das Clínicas, Faculdade de Medicina, Universidade de São Paulo (USP), São Paulo, SP, Brazil; g Department of Surgery, Faculdade de Medicina, Universidade de São Paulo (USP), São Paulo, SP, Brazil; h Department of Health, Hospital Heliópolis, São Paulo, SP, Brazil; i Hospital São José da RBBP, São Paulo, SP, Brazil

**Keywords:** Neurilemmoma, Neurofibromatosis, Neurofibromatoses, Neurofibrosarcoma, Head and neck neoplasms, Neurilemoma, Neurofibromatose, Neurofibromatoses, Neurofibrossarcoma, Neoplasias de cabeça e pescoço

## Abstract

**Introduction:**

Peripheric nerve tumors typically derive from Schwann cells of the peripheral nerve sheet. Since these tumors are uncommon, they should be considered in preoperative differential diagnosis.

**Objective:**

To report the experience of a tertiary care department.

**Methods:**

Forty-two patients with head and neck peripheral neurogenic tumors were retrospectively analyzed and evaluated from 1977 to 2013. The preoperative diagnosis was confirmed by biopsy or imaging study.

**Results:**

The mean age was 41.7 and 15 patients (36%) were male. The mean size was 5.5 cm and 26 (61%) were located laterally in the neck. Most tumors (39.9%) presented as an asymptomatic neck mass. Most (39.9%) were resected through a neck approach. Cranial nerves were the commonest site of origin.

**Conclusions:**

Extracranial neurogenic tumors presented with a mean size of 5.5 cm, were located laterally in the neck, normally had their origin from cranial nerves, and their resection approach is cervical.

## Resumo

**Introdução:**

Tumores dos nervos periféricos tipicamente derivam das células de Schwann da bainha dos nervos periféricos. Por serem incomuns, devem ser lembrados no diagnóstico diferencial pré-operatório.

**Objetivo:**

Relatar a experiência de serviço de referencia terciária.

**Método:**

De 1977 a 2013, 42 pacientes com tumores neurogênicos periféricos da cabeça e pescoço foram operados e analisados retrospectivamente. A confirmação diagnóstica pré-operatória deu-se por biópsia ou método de imagem.

**Resultados:**

A média da idade foi de 41,7 anos, sendo 15 indivíduos (36%) do gênero masculino. O tamanho médio foi de 5,5 cm e 26 (61%) localizavam-se na face lateral do pescoço. A maior parte (39,9%) apresentou-se como tumor palpável assintomático. A maioria (39,9%) foi ressecada por acesso cervical. A maioria originou-se de nervos cranianos.

**Conclusões:**

Tumores neurogênicos extracranianos apresentam-se com tamanho médio de 5,5 cm, na face lateral do pescoço, costumam originar-se de nervos cranianos e ser ressecados por via cervical.

## Introduction

Tumors arising from peripheral nerves typically are derived from Schwann cells located in the peripheral nerve sheath, commonly from cranial nerves, but also from sensory or motor nerves and nerves of the sympathetic nervous system. Among the many names used to describe these tumors, two in particular – schwannomas and neurofibromas – have significant clinical differences that require discussion. As a group, neurogenic tumors occur most commonly in the head and neck in 25–45% of cases.^1^, ^2^, ^3^

These tumors are reported in the parapharyngeal and retropharyngeal space, posterior pharyngeal wall, paranasal sinuses, nasal cavity, scalp, submandibular region, larynx, epiglottis, tongue, infratemporal fossa, oral cavity, etc.^4^, ^5^, ^6^ Often, such tumors present as an asymptomatic lateral neck mass, although they can cause symptoms such as nasal obstruction, dysphagia, and dysphonia, depending on their location and size. Sometimes these tumors may be associated with other diseases, such as multiple endocrine neoplasia or neurofibromatosis type 1. Considering that they are relatively rare, these tumors should be considered in preoperative differential diagnosis, as other primary neck tumors may present as an asymptomatic neck mass; moreover, their resection may require neural reconstruction, and the surgeon must be prepared for this possibility.

The aim of this study was to report the experience in addressing these tumors by a tertiary referral service.

## Methods

This study was approved under No. 554 by the Ethics Committee of the institution.

**Figure 1:**
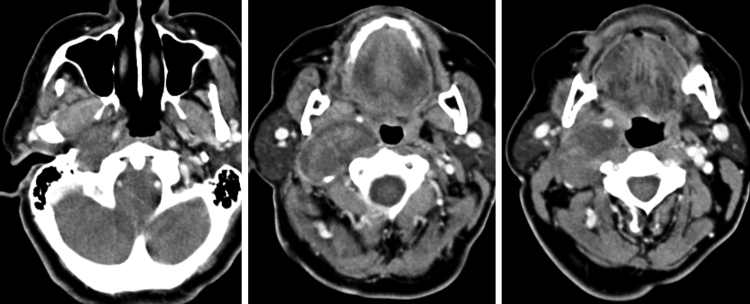
Valgus nerve schwannoma: axial computed tomography shows a heterogeneous mass with well-defined limits, displacing the internal carotid artery and internal jugular vein.

**Figure 2:**
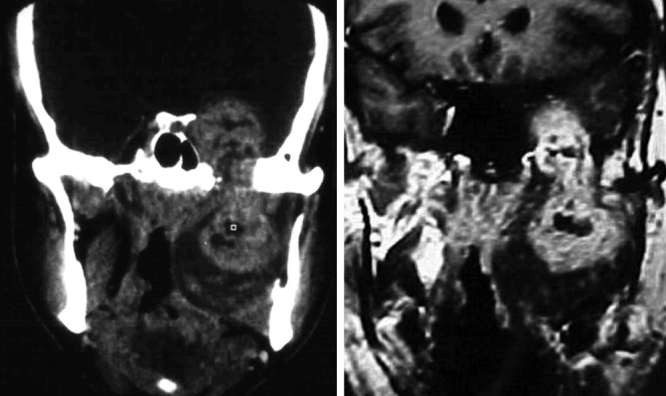
Mandibular nerve schwannoma: coronal computed tomography and magnetic resonance imaging show a hourglass-like mass with a localized shaft and widening of the oval foramen, with intracranial components and in the masticator space.

**Figure 3:**
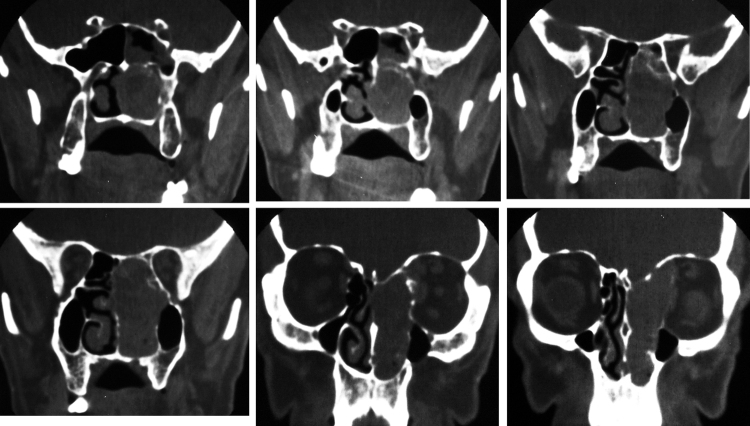
Naso-ethmoid neurofibroma: coronal computed tomography with soft tissue window, showing a naso-ethmoidal solid mass with medial orbital wall remodeling.

From December of 1977 to December of 2013, 42 patients with neurogenic tumors of the head and neck with peripheral origin underwent surgery. The medical records of these patients were retrospectively reviewed. After history collection and physical examination, the collection of material for histopathological diagnosis was performed whenever possible, for diagnostic purposes. Thus, in tumors that could be accessed at physical examination or by endoscopy, such as nasal and oral cavity tumors, incisional biopsy or puncture was performed. Fine needle aspiration (FNA) was performed on tumors located in more superficial portions of the neck. In the case of deep tumors, such as those arising in the parapharyngeal space (one case), tissue collection could not be performed (Table 1). In many patients, computed tomography (CT) and/or magnetic resonance imaging (MRI) were performed (Figure 1, Figure 2, Figure 3).

**Table 1:** Anatomic site of 42 head and neck neurogenic tumors and diagnosis modality.

Location	*n* (%)	Diagnostic by imaging method	Diagnostic by FNA	Diagnosis by incisional biopsy
Lateral	26 (61)	4	16	6
Supraclavicular fossa	7 (16)	1	1	5
Submandibular	4 (9)	0	1	3
Integument	3 (7)	0	1	2
Parotid gland	1 (2)	0	1	0
Frontoethmoidal region	1 (2)	1	0	0

FNA, fine needle aspiration.

Tumor excision (with separation of the nerve of origin) and intracapsular enucleation in cases of schwannoma, and resection with a safety margin in case of neurofibromas, were conducted. Histological diagnosis was confirmed with the study of paraffin block specimens (Table 2).

**Table 2:** Histopathologic types.

Histology	*n* (%)
Schwannoma	20 (48)
Neurofibroma	18 (43)
Neurofibromatosis	3 (7)
Neurosarcoma	1 (2)

## Results

At treatment, the mean age was 41.7 years (range 12–77 years). Fifteen patients (36%) were male and 27 (64%) female. The mean tumor size was 5.5 cm (range 2.0–15.0 cm). Of these tumors, 26 (61%) occurred in the lateral aspect of the neck (Table 1).

Most tumors (39.9%) presented as a palpable asymptomatic mass. Two (5%) patients presented with paresthesia in upper limb, and one case (2%) had pain and obstructive symptoms.

Most tumors (39.9%) were resected by cervical access. One patient presented bulging in the hemiface and signs of nasal obstruction, and underwent a biopsy via Caldwell-Luc procedure and subsequent partial maxillectomy. In both cases, the resection was carried out by intracapsular enucleation. In one of the cases, referred with previous diagnosis of lateral cervical neurofibroma, the CT showed bone erosions in C4 and C5 vertebral bodies, and the patient underwent resection of the lesion with origin in C4 and C5 roots, requiring resection of parts of the vertebral body and subsequent reconstruction. There was no recurrence in this patient population after a follow-up ranging from 24 to 60 months (mean 40 months).

As for the nerve of origin, most tumors originated from cranial nerves VII, X, XI, and XII, ten cases stemmed from the brachial plexus, followed by cervical plexus, cervical sympathetic, C3 to C7 roots; and in five cases their origin was not identified (Table 3).

**Table 3:** Tumor origin.

Origin	*n* (%)
Brachial plexus	10 (24)
Cervical plexus	9 (21)
Vagus nerve	4 (10)
C3 to C7 roots	4 (10)
Cervical sympathetic	3 (7)
Lingual nerve	3 (7)
Hypoglossal nerve	2 (5)
Accessory nerve	1 (2)
Facial nerve	1 (2)
Minor nerves/not defined	5 (12)

## Discussion

Embryologically, neurogenic tumors originate from the neural crest and can be formed from Schwann cells or synpathoblasts.^7^ The former cells originate from a specialized population of neuromesenchymal cells from the neural crest, giving rise to schwannomas and neurofibromas.^8^ These two entities can arise from any cranial nerve or spinal root with a sheath, that is, from any motor or sensory nerve, except the optic and olfactory nerves, which have no Schwann cell sheath, as they are direct extensions of the central nervous system.^9^

The cell population of schwannomas has an exclusive origin from Schwann cells, while neurofibromas comprise a mixture of three cells: Schwann cells, perineural cells, and perineural fibroblasts.^8^

In the present study, the mean age of patients was 41.7 years, with predominance of females (27/42); some authors agree^1^ with this finding, while others disagree.^10^ A study reported that schwannomas can arise at any age, with no preference for age or race.^7^

The size of these tumors can range from a few millimeters to over 24 cm.^7^ In this sample, the variation was 2–15 cm with a mean of 5.5 cm.

In general, the schwannoma is a slow-growing, isolated, encapsulated tumor, connected with the nerve of origin. When localized in the head and neck, it affects patients at any age; however, reports indicate a preponderance in the fifth decade of life.^11^ Generally, schwannomas show degenerative changes such as cystic alterations and hemorrhagic necrosis; such changes are not seen in neurofibromas.^2^ If there is involvement of a major nerve, there may be severe dysfunction that, clinically, can manifest as vocal fold paralysis, Horner syndrome, or sensory or motor dysfunction of the upper limb, among others. In the present study, two (2%) patients had sensory disorder of the upper limb, one (1%) patient had obstructive symptoms, and the remaining presented a neck mass; this coincides with the literature.^7^

According to reports in the literature, the most common site of extracranial schwannomas in the head and neck is the parapharyngeal region: in fact, these are usually retro-styloid injuries in the carotid space that cause anterior displacement of the fat present in the pre-styloid parapharyngeal space, with bulging of the lateral wall of oropharynx.^12^, ^13^, ^14^, ^15^ Other sites in the head and neck, such as the submandibular region, paranasal sinuses, face, and oral cavity, are rare.^16^ As for location, cases on the lateral aspect of the neck predominated, and most occurred in the parapharyngeal region.

Most often, neurofibromas affect patients aged 20–30 years and have no predilection for gender. Clinically, neurofibromas can present as an isolated tumor or in a multiple form (disseminated tumors). Most often, localized neurofibromas arise from cutaneous nerves, with occasional involvement of deep nervous sheath. This is a benign, slow-growing, relatively circumscribed but unencapsulated tumor. In the first case, there is no known cause (60–90% of cases), and these tumors are known as solitary neurofibroma, with varying anatomic distribution.^17^ In about 10% of cases, there is an association with neurofibromatosis type 1 syndrome, in association with a somatic mutation in the NF1 gene, a tumor suppressor gene located on chromosome 17.^18^

A critical issue regarding the treatment of neurogenic tumors of the head and neck is the diagnosis, which is based on clinical findings, namely, the onset of a long-standing cervical mass. However, these tumors can also be discovered incidentally on imaging studies. The determination of the nerve of origin is strategic, since this provides the possibility of an informed decision, by the patient, about any risk of post-treatment functional sequelae.Although cytology may be of assistance, in most cases the technique of aspiration puncture is inconclusive.^19^, ^20^, ^21^

The specificity of imaging studies is also not high. These tests lend themselves more to treatment planning and evaluation of the vascularization of tumors, which is sometimes very rich, and are less useful for diagnosis of the tumor's nerve of origin, although that is possible in certain situations.^22^ As for the use of CT and MRI, most authors prefer the latter technique.^23^, ^24^ High-resolution CT (HRCT) determines the size and extent of the tumor, demonstrates the degree of tumor vasculature, and differentiates between benign and malignant lesions. In CT and MRI, schwannomas usually present as a spherical or ovoid soft tissue mass, and can show non-homogeneous contrast enhancement, a cystic component, and fatty degeneration.^25^

Incidence of malignant schwannomas and the malignant transformation rate of benign schwannomas are not available in the literature. A study on intracranial malignant peripheral nerve sheath tumors suggested sporadic development, and no transformation of benign tumors.^26^ Most neurofibromas are single lesions not associated with neurofibromatosis; however, when combined with NF1, there is a small risk of malignancy. Based on this circumstantial evidence, in both cases the malignant transformation rate can be considered a rare event in patients with solitary schwannoma and neurofibroma of the head and neck. Because of the indolent nature and the remote chance of malignant transformation, it is possible to opt for clinical follow-up. Therefore, the decision in favor of surgery should be based on an analysis of the risks and benefits of the surgical treatment; i.e., preoperative symptomatic severity and predicted postoperative neurological deficit.

Among the differential diagnoses, reactive or metastatic lymph node diseases; soft tissue tumors such as fibroma, leiomyoma, lipoma, and paraganglioma; carotid artery aneurysm; and branchial abnormalities should be included.

The choice between resection of the tumor with sectioning of the nerve of origin or intracapsular enucleation with the possibility of preserving nerve function is mainly determined by the relationship between the tumor and the nerve of origin. Intracapsular enucleation is more feasible in eccentrically located tumors, without dispersing the neural fibers. In neurofibromas, the rule is to perform tumor resection with a safety margin.

In histopathological examination, the classic schwannoma reveals a biphasic pattern composed of two types of cellular patterns: Antoni A and Antoni B. Antoni A areas are composed of spindle cells with long, thin fibers, forming nuclear arrangements in parallel, known as palisading nuclei, around a central cytoplasmic mass (Verocay bodies). Antoni B areas are of degenerative nature. The tissue is a loosely disposed stroma in which neural fibers and cells do not form any distinct pattern. Both types can arise together. Variants of schwannoma were described; cellular, plexiform, epithelioid, melanotic, and ancient forms are the five main variants of this tumor. Three variants of neurofibroma have also been described: localized, plexiform, and diffuse forms.

The recurrence rates after resection are not well documented in the literature. Recurrence following *en bloc* resection is a rare finding. In the present series, there was no recurrence after a mean follow-up of 40 months. The tumor is radio-resistant, and radiation therapy should be reserved for palliative treatment in cases where surgery is impossible, however imperative.^3^

## Conclusions

The mean age was 41.7 years; 36% were male. Mean size was 5.5 cm; 61 tumors were localized in the lateral aspect of the neck; 39.9% presented as a palpable asymptomatic mass; 39.9% were resected through cervical access and most of these lesions had their origin in the cranial nerves.

## Conflicts of interest

The authors declare no conflicts of interest.
